# The Role of Non-Coding RNAs in Epigenetic Dysregulation in Glioblastoma Development

**DOI:** 10.3390/ijms242216320

**Published:** 2023-11-14

**Authors:** Ekaterina Isachesku, Cornelia Braicu, Radu Pirlog, Anja Kocijancic, Constantin Busuioc, Lavinia-Lorena Pruteanu, Deo Prakash Pandey, Ioana Berindan-Neagoe

**Affiliations:** 1 Research Center for Functional Genomics, Biomedicine and Translational Medicine, Iuliu Hatieganu University of Medicine and Pharmacy, 400337 Cluj-Napoca, Romaniacornelia.braicu@umfcluj.ro (C.B.); pirlog.radu@yahoo.com (R.P.); pruteanulavinia@gmail.com (L.-L.P.); 2 Department of Microbiology, Oslo University Hospital, 0424 Oslo, Norway; akocijancic@gmail.com (A.K.);; 3 Department of Pathology, National Institute of Infectious Disease, 021105 Bucharest, Romania; busuioc.constantin@gmail.com; 4 Department of Pathology, Onco Team Diagnostic, 010719 Bucharest, Romania; 5 Department of Chemistry and Biology, North University Center, Technical University of Cluj-Napoca, 430122 Baia Mare, Romania

**Keywords:** glioblastoma, epigenetics, miRNAs, ncRNAs

## Abstract

Glioblastoma (GBM) is a primary brain tumor arising from glial cells. The tumor is highly aggressive, the reason for which it has become the deadliest brain tumor type with the poorest prognosis. Like other cancers, it compromises molecular alteration on genetic and epigenetic levels. Epigenetics refers to changes in gene expression or cellular phenotype without the occurrence of any genetic mutations or DNA sequence alterations in the driver tumor-related genes. These epigenetic changes are reversible, making them convenient targets in cancer therapy. Therefore, we aim to review critical epigenetic dysregulation processes in glioblastoma. We will highlight the significant affected tumor-related pathways and their outcomes, such as regulation of cell cycle progression, cell growth, apoptosis, angiogenesis, cell invasiveness, immune evasion, or acquirement of drug resistance. Examples of molecular changes induced by epigenetic modifications, such as DNA epigenetic alterations, histone post-translational modifications (PTMs), and non-coding RNA (ncRNA) regulation, are highlighted. As understanding the role of epigenetic regulators and underlying molecular mechanisms in the overall pro-tumorigenic landscape of glioblastoma is essential, this literature study will provide valuable insights for establishing the prognostic or diagnostic value of various non-coding transcripts, including miRNAs.

## 1. Introduction

Glioblastoma (GBM) is a highly aggressive form of brain cancer that arises from glial cells, primarily astrocytes. It is the most common and deadliest type of primary brain tumor in adults. The annual incidence rate is estimated to be around 3 cases per 100,000 people [1,2,3].

GBMs are classified into two main categories based on the presence or absence of specific genetic mutations. The primary (or de novo) GBMs arise without any prior history of lower-grade gliomas, while secondary GBMs develop from lower-grade gliomas that progress into higher-grade tumors [4].

GBM is a highly heterogeneous disease. Tumor heterogeneity translates into histological patterns and epigenetic, genetic, and transcriptomic alterations [4,5]. GBMs tend to proliferate and infiltrate surrounding brain tissue, making them difficult to treat effectively. Standard GBM treatment typically involves a combination of surgery, radiation therapy, and chemotherapy [6]. Surgical resection aims to remove as much of the tumor as possible, while radiation therapy and adjuvant chemotherapy (with the alkylating agent temozolomide) target the remaining cancer cells [7]. Despite significant advances in GBM diagnosis and therapy, an essential role in therapy resistance is attributed to glioma stem cells (GSCs). They represent slow-dividing cells with self-renewal properties [8]. In most cases of malignant recurrence, GSCs are responsible for tumor regrowth, supporting GBM heterogeneity and playing a crucial role in regulating the tumor microenvironment (TME) homeostasis. Study results indicate that radiotherapy resistance and active populations of GSC are positively correlated [9,10,11]. As ionizing radiation (IR) provokes different DNA damage, repairing mechanisms are activated for cellular survival. Besides a dysregulated genetic profile, which frequently includes molecular alterations in *TP53*, *ATRX*, *IDH1/2*, *CDKN2A-p16*, *PTEN*, or *EGFR* genes, epigenetic dysregulations also play a significant role in the modulation of chemoresistance and should be explored as a potential tool for developing new treatment approaches [12,13].

Epigenetic markers are crucial in GBM, providing insights into disease progression, prognosis, and potential therapeutic targets. Understanding the epigenetic alterations associated with GBM has significant implications for diagnosis, prognosis, and the development of targeted therapies. Researchers are actively investigating various epigenetic modifications and their roles in GBM, aiming to potentially develop epigenetic-based treatments to improve patient outcomes. These changes can affect the expression of genes involved in crucial cellular processes such as cell growth, DNA repair, and cell death. In GBM, as in many other cancers, epigenetic changes are influencing the development and progression of the disease. Depending on the mechanisms that modulate the epigenetic machinery, these changes can be consequential to some genetic events that are influencing the functions and roles of epigenetic modifiers [14,15]. Other mechanisms, such as O^6-methylguanine-DNA methyltransferase (MGMT) hypermethylation, have a causative role, as this epigenetic modification is an important recurrent epigenetic event in GBM that is a factor responsible for chemotherapy resistance, its evaluation being routinely conducted in the clinic [15]. Also, the epigenetic changes can impact the regulation of GSCs.

There are four well-known epigenetic modifications: DNA epigenetic alterations, histone post-translational modifications (PTMs), non-coding RNAs (ncRNAs), and remodeling chromatin complexes [16].

One of the most common DNA epigenetic modifications is DNA methylation, which involves adding a methyl group to the DNA molecule. Aberrant DNA methylation patterns have been observed in GBM cells, leading to the silencing of tumor-suppressor genes and activating oncogenes. These changes can promote uncontrolled cell growth and tumor formation. Hypermethylation of specific gene promoter regions can lead to silencing tumor-suppressor genes, such as MGMT. The MGMT promoter methylation status is a prognostic and predictive marker in GBM.

Histone modifications are another critical aspect of epigenetic regulation. Histones are proteins around which DNA is wrapped, forming a structure called chromatin. Different modifications to histone proteins, such as methylation, acetylation, and phosphorylation, can influence gene expression, as these modifications can affect the packaging of DNA into a condensed form, making specific genes accessible for transcription. In GBM, histone PTMs have been implicated in the dysregulation of gene expression networks that drive tumor progression.

Furthermore, ncRNAs, particularly microRNAs, have become crucial players in GBM development [17,18,19]. miRNAs are small, ncRNA molecules critical in regulating gene expression. They are involved in various biological processes, including development, cell proliferation, differentiation, and apoptosis (programmed cell death). miRNAs bind to messenger RNA (mRNA) molecules, which are the templates for protein synthesis, either degrading or inhibiting their translation into proteins [19,20,21]. Abnormal expression of microRNAs in GBM can disrupt the regulation of target genes involved in cancer-related processes.

Finally, chromatin remodeling complexes are ATP-dependent multiprotein machinery that manipulates the chromatin state by changing the nucleosome structure and/or composition. In the context of GBM, these complexes play a role in the dysregulation of gene expression patterns, contributing to tumor initiation, progression, and therapeutic resistance [22]. Dysregulation of SWI/SNF (SWItch/Sucrose Non-Fermentable) chromatin remodeling complex components has been linked to GBM pathogenesis. GBM cases with SWI/SNF alterations often exhibit unique DNA methylation patterns and transcriptional profiles [22]. These alterations can serve as prognostic markers or therapeutic targets in the future. Like the SWI/SNF complex, the INI1/hSNF5 complex alterations can result in abnormal chromatin structure and dysregulated gene expression patterns [23]. Other investigations revealing the role of remodeling complexes in brain cancer have also been described in the literature [24,25,26]. However, more studies can be required to elucidate the subtleties of the underlying mechanisms of these epigenetic elements in regulating GBM development.

Moreover, the approach of epigenomic editing holds the potential to selectively modify and regulate epigenetic marks, providing a targeted approach to alter gene expression patterns. Epigenomic editing techniques, such as CRISPR-based technologies, could be employed to selectively remove or modify repressive epigenetic marks, aiming to reactivate these tumor-suppressor genes and inhibit tumor growth [27]. CRISPR/Cas9 has emerged as a potent tool for precise genome editing; in the context of GBM, CRISPR/Cas9 was used to induce specific genetic alterations, such as the simultaneous deletion of TP53, PTEN, and NF1, in mice. This approach aimed to mimic the genetic landscape associated with glioblastoma development, providing valuable insights into the role of these genes in tumor initiation and progression [27]. Another study was focused on the CDKN2A gene, which encodes the p16INK4a protein, often inactivated in glioblastoma [28].

Currently, some epigenetic-modifying drugs such as vorinostat, panobinostat, romidepsin and valproic acid are being tested in clinical trials with classic treatment strategies such as TZM and radiotherapy [29]. Epigenomic editing could be used to introduce repressive marks or modify the epigenetic landscape around oncogenes, leading to their inactivation. This could help suppress the proliferative capacity of glioblastoma cells.

## 2. The Interplay between DNA Epigenetic Alterations and miRNA Regulation in GBM

The intricate interplay between DNA epigenetic alterations and the regulatory process of miRNAs in GBM paints a vivid picture of molecular complexity. In GBM, DNA epigenetic alterations, such as aberrant methylation patterns and modifications, act as silent architects, reshaping the landscape of gene expression without altering the underlying genetic code.

### 2.1. DNA Methylation and miRNA

Several DNA epigenetic alterations can serve as tumorigenesis starting points, such as DNA methylation, demethylation, hydroxymethylation, and other oxidation derivatives. DNA methylation is an epigenetic modification involving adding a methyl group to DNA molecules, often occurring at CpG dinucleotide sites. As about 40–60% of CpG islands are found in gene promoters, added methyl groups block their binding with transcription factors. Therefore, DNA methylation is recognized as a gene repression/silencing mark. Alterations in the DNA methylation machinery contribute to the aberrant DNA methylation patterns observed in various tumor cells, including those of GBM. DNA methyltransferases (DNMTs) are enzymes that add methyl groups to DNA. DNMT1, DNMT3A, and DNMT3B are the main DNMTs in establishing and maintaining DNA methylation landscape. Dysregulation of DNMTs can result in abnormal DNA methylation patterns in GBM.

For example, Zhang and colleagues reported differential methylation levels when comparing the whole genome of primary glioma with that of non-tumor brain tissues [30]. They identified 524 hypermethylated and 104 hypomethylated areas, among which 361 hypermethylated and 70 hypomethylated regions were CpG islands. The genes with a hypomethylated promoter regulate the apoptotic program, transcription factors and DNA binding, nervous system development, neuroactive ligand–receptor interaction, and other processes [30]. The importance of methylation status in glioma led to establishing the Glioma CpG island methylator phenotype (G-CIMP), which provides clinically relevant information regarding further glioma classification independent of grade and histology [31].

Moreover, methylation signature is not specific only for common tumor-suppressor genes but also for methyltransferases. O6-methylguanine-DNA methyltransferase (MGMT) is a DNA “suicide” repair enzyme which transfers the methyl group from the O^6^ site of guanine to its cysteine residue. The process leads to its irreversible inhibition while activating DNA repair mechanisms and supporting increasing chemoresistance. As mentioned earlier, temozolomide (TMZ) is a cytotoxic alkylating agent used in GBM treatment in adjuvant chemotherapy. It acts opposite to MGMT by adding methyl groups to the N7 and O^6^ guanine and the N3 adenine positions. Despite promising effects, the main issue of TMZ resistance acquirement is reported [32,33]. In this regard, MGMT promoter methylation status is a broadly studied prognostic biomarker in GBM, as its unmethylated form and MGMT overexpression are some of the most important causes of TMZ resistance. It is correlated with a decreased survival rate of GBM patients as they become less sensitive to the treatment [34]. However, some studies show a negative correlation between MGMT promoter methylation status, gene expression level, and patient outcome, indicating that MGMT is not expressed despite the lack of promoter methylation in some GBM cases. Given this, the biomarker status of MGMT methylation remains under debate [35,36].

In their turn, DNMTs are found to be regulated by other epigenetic components—miRNA molecules. By modulating DNA methylation machinery gene expression, miRNAs can significantly affect cellular processes and contribute to various epigenetic dysregulation in glial cells, providing different outcomes in each case. As such, it was demonstrated that **miR-152-3p** acts as a tumor suppressor by regulating glioma cell viability. The results suggested that a decreased level of the miR-152-3p correlated with an increased level of DNMT1, confirmed by forced overexpression of the miRNA and direct inhibition of the enzyme’s expression on the mRNA level. Furthermore, reduced methyltransferase activity was linked with the hypomethylated status of NF2 promoter and a significant increase in cell apoptosis and a decrease in cell invasion compared with the control [37]. Another RNA targeting DNMT1 is **miR-185**. Transfection of glioma cells with miR-185 significantly reduced the levels of DNMT1 mRNA transcripts and did not affect the expression of DNMT3A and DNMT3B. Moreover, it was established that the miRNA targets DNMT1 mRNA at the 3′-UTR [30].

**MiR-29c** is a direct inhibitor of the de novo DNA methyltransferases **DNMT3a/DNMT3b** and is an indirect suppressor of MGMT transcription factor. By restoring the expression level for this transcript, which is underexpressed in GBM, it was possible to sensitize cells to TMZ [38]. **MiR-129-5p** was found to target DNMT3A, and overexpression of this miR could inhibit human glioma cell proliferation and induce cell cycle arrest [39].

Interconnections between epigenetic components are intricate and do not work unidirectionally. Given this, promoters of some miRNAs, which act as tumor suppressors, are also hypermethylated, leading to their downregulation and establishment of an advantageous landscape for activating pro-tumorous pathways. As in the case of **miR-148a**, a comprehensive study indicated that its hypermethylation and silencing were correlated with a mutant profile of the isocitrate dehydrogenase (IDH1^mut^) gene after it was compared with a wild-type IDH1^WT^. Usually, IDH1 participates in the tricarboxylic acid cycle, where it metabolizes isocitrate to α-Ketoglutarate (α-KG), an essential cofactor for specific histone and DNA demethylases. Consequently, a mutation in IDH1 is always linked to disruption of cell metabolism and alteration of the epigenetic landscape, as it contributes to the DNA hypermethylation pattern observed in G-CIMP. Further analysis evidenced tumor-suppressive features of miR-148a by miR overexpression and assessment of significant G0/G1 cell cycle arrest in 2 glioma cell lines [40,41,42].

Methyl-CpG-Binding Proteins (MBPs) are proteins that specifically bind to methylated DNA regions, altering the gene expression in various conditions and GBM. For example, it was demonstrated that methyl-CpG-binding domain protein 2 (MBD2) increases concentration in GBM cells. Further analysis showed that this event led to downregulation of brain angiogenesis inhibitor 1 (BAI1) via binding to its hypermethylated gene promoter [43].

### 2.2. DNA Demethylation and miRNAs

On the other side, DNA demethylation is the process that could counterbalance the occurring hypermethylated pattern in tumorous cells. It is well known that methyl groups can be removed by Ten–eleven Translocation (TET) enzymes [44,45]. TET is involved in the active demethylation process by oxidizing 5-methylcytosine (5mC). TET enzymes interact with other proteins and signaling pathways implicated in GBM. For example, TET1 can physically interact with the EGFR protein, a commonly amplified and mutated gene in GBM. This interaction may influence the downstream signaling of EGFR and impact tumor growth and resistance to therapy.

In the study conducted by Forloni et al., the relationship between oncogenic EGFR and anti-oncogenic **TET1** was described. It was suggested that overexpressed EGFR inhibits TET1 activity via the C/EBPα transcription factor. As a result, *TET1* repression was associated with the hypomethylation of tumor-suppressor genes and EGFR-mediated epigenetic silencing [46].

Another study confirmed the correlation between TET1 activity loss, low enzyme mRNA levels, and EGFR amplification. Additionally, after performing whole-exome sequencing-based analysis, the minimal deleted region at chromosome 10 was detected at the DNA demethylase TET1, mainly due to loss of heterozygosity of complete chromosome or monoallelic microdeletion. Also, incidences of TET1 deletion were significantly dropped in patients with mutated IDH1. These findings confirm the intricate bidirectional interplay between genetic mutations and dysregulated epigenetic disorders [47].

Specifically, **TET2** is often mutated or silenced, leading to a global decrease in DNA hydroxymethylation, an intermediate step in the DNA demethylation process. In vitro data suggested a reduction in cell viability of GBM cell line LN229 after ectopic overexpression of TET2. In vivo studies showed that nude mice with TET2-transfected LN229 cells presented much lower tumorigenic potential with a considerable difference in tumor weight. Moreover, altered expression of 19 neuroectodermal markers in TET2-overexpressed cells was determined, among which the most significant changes were observed for Mash1 and Cystathionine β-synthase [48,49]. TET2 loss is associated with stem cell features and correlates with poor survival of patients with GBM [50].

An important role in TET2 regulation was attributed to **miR-10b-5p**. Their expression levels were found to be inversely correlated in GBM tissues. Further investigation showed that *TET2* overexpression and miR-10b-5p inhibition led to PD-L1 activity loss, leading to deregulation in cell aggressiveness and immune evasion. Analysis suggested that the possible underlying mechanism depends on the recruitment of histone deacetylases (HDAC1 and HDAC2) to PD-L1 promoter by TET2, as occupancy of the promoter site of these three enzymes was demonstrated. This study represents a valuable example that elucidates possible mechanisms of interconnections between multiple epigenetic components and their contribution to the overall cellular responding [51].

Understanding TET enzymes’ role in GBM is crucial for developing targeted therapies. Modulating TET enzyme activity or restoring normal DNA methylation patterns represents a potential therapeutic strategy. However, further research is needed to fully elucidate the complex mechanisms underlying TET enzyme dysregulation in GBM and to translate this knowledge into effective treatment options.

**Figure 1:**
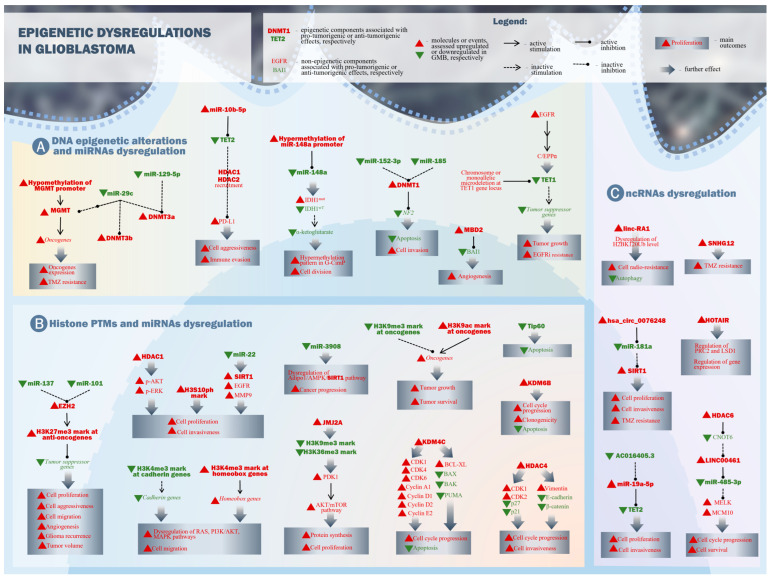
Epigenetic dysregulation in glioblastoma. (**A**) Major DNA epigenetic alterations with some correspondent miRNAs dysregulated levels are presented. Driver changes include upregulation of pro-tumorigenic (DNMT1, DNMT3a, DNMT3b, miR-10b-5p, MBD2), as well as downregulation of anti-tumorigenic (TET1, TET2, miR-29c, mR-129c-5p, miR-148a, miR-152-3p, miR-185) epigenetic elements. (**B**) Histone PTMs and certain miRNAs play a role in their regulation. Histone PTMs comprise altered elements of histone methylation (EZH2, H2K27me3 mark, H3K4me3 mark, H3K9me3 mark), demethylation (KDM4C, KDM6B, JMJ2A), acetylation (Tip60), deacetylation (HDAC1, HDAC4, SIRT1), and others (such as H3S10ph or H2BK120ub marks). Like in the case of DNA alterations, the role of histone PTM elements is suggestively depicted in red or green colors. It is worth noting that the same histone mark, like H3K4me3, can play a dual pro- or anti-tumorigenic role depending on the location within the genome. (**C**) ncRNAs, other than miRNAs, also contribute to epigenetic landscape modulation, usually by binding and inhibiting other target epigenetic modulators. LncRNAs (HOTAIR, linc-RA1, SNHG12, LINC00461, AC016405.3) and circRNAs (has_circ_0076248) were found to be involved in certain molecular pathways, affecting the progression of GBM. Usual outcomes comprise oncogene expression, cell proliferation, immune evasion, cell invasion, and others listed in the boxes for specific altered pathways.

In GBM, specific miRNAs, identified as epigenetic markers related to epigenetic DNA alterations, are summarized in Table 1. A schematic representation of epigenetic alteration at the DNA level and related miRNAs, as well as triggering effects of dysregulations in GBM, are depicted in Figure 1A.

## 3. Relations between Histone PTMs and miRNAs in GBM

Histone PTMs, including methylation, acetylation, phosphorylation, and ubiquitination, influence gene expression in GBM [52]. Understanding the specific histone modifications can provide insights into the disease mechanisms and potentially guide the development of targeted therapies to restore normal chromatin regulation [45]. Below are just a few examples of the histone modifications implicated in GBM.

### 3.1. Histone Methyltransferases and miRNAs

Like DNA epigenetic alteration, methyl groups can be added to histone proteins, which further affects the conformational availability of chromatin, histone methyltransferases (HMT) being enzymes responsible for this process. EZH2 is a polycomb-repressive complex 2 (PRC2) component involved in epigenetic regulation [53]. EZH2 acts as a histone methyltransferase and adds methyl groups to histone proteins, leading to gene silencing [54]. It plays a role in GBM by promoting tumor growth and invasion. EZH2 overexpression can lead to the repression of tumor-suppressor genes and activation of oncogenes, contributing to GBM progression [53,54]. Consequently, an increased expression of EZH2 has been observed in GBM tumors, and it correlates with poor patient prognosis [53].

There is evidence to suggest that miRNAs can regulate **EZH2** expression in GBM. Specific miRNAs, like miR-101-3p and miR-137, have been identified as direct or indirect regulators of EZH2 [54]. For example, some miRNAs can directly target the EZH2 mRNA, leading to its degradation or blocking its translation into protein. On the other hand, specific miRNAs can indirectly regulate EZH2 by targeting other molecules involved in its regulation. Therefore, by modulating EZH2 expression, miRNAs can influence GBM cells’ epigenetic landscape and gene expression patterns.

In the study conducted by Smits and colleagues, an elevated level of EZH2 was confirmed in GBM cases, which were compared with non-neoplastic brain cells after performing an immunohistochemistry assay. Increased enzyme expression was also positively correlated with glioma aggressiveness according to glioma grade and glioma recurrence data analysis. Furthermore, the contribution of miR-101 in EZH2 regulation was tested, accomplishing transfection of human U87 GBM cells with pre-miR-101, EZH2 siRNA, and DZNep, an inhibitor of S-adenosylhomocysteine hydrolase. In all three cases, EZH2 protein expression and the evaluated level of H3K27 trimethylation were significantly reduced. The binding of miR-101 to the EZH2 3′-UTR at two sites was described as a possible mechanism of direct EZH2 inhibition. Further, in vitro experiments presented considerable alteration in cellular proliferation and migratory and angiogenetic abilities, while in vivo experiments revealed that tumors’ volume has been drastically reduced in the EZH2-inhibited group versus the control group [54].

**MiR-137** is another example of miRNA with significantly downregulated expression in glioma tissue, according to TCGA datasets and qRT-PCR validation analyses, which contribute to alterations of histone methylation patterns. miR-137 mimic transfection into U87 cells had similar effects as those induced by EZH2 inhibition [55].

The interplay between miRNAs and EZH2 in GBM is complex and multifaceted. Dysregulation of miRNAs can contribute to GBM development and progression, while EZH2 overexpression can affect gene expression through epigenetic mechanisms. Understanding the regulatory relationships between miRNAs and EZH2 in GBM may provide insights into the underlying biology of the disease and potentially open avenues for novel therapeutic strategies, such as utilizing miRNAs to modulate EZH2 or targeting EZH2 directly as a therapeutic intervention.

Nonetheless, it is crucial to understand that consequent methylation performed by the enzyme can lead to different outcomes, as several added methyl groups, as well as the position of methylated amino acids, play significant roles in conferring either condensed or relaxed chromatin state, which affects the availability of promoter for TFs bindings. For example, trimethylation of H3K4 is associated with activation of gene expression, while H3K9me, H3K27me, and H4K20me marks have the opposite effect [56].

Altered levels of H3K4 methylation have been observed in GBM. **H3K4me** is involved in transcriptional activation, and both increased and decreased levels of H3K4me have been reported in different studies, suggesting complex regulatory mechanisms in GBM.

Studies have shown increased and decreased lysine 4 trimethylation levels of histone H3 (**H3K4me3**) in GBM compared to normal brain tissue [57,58]. These changes can affect the expression of genes involved in critical cellular processes, including cell cycle regulation, DNA repair, and tumor suppression. For example, a global reduction of H3K4me3 was demonstrated in GBM samples compared with GBM-surrounding tissues after performing NGS analysis. The results indicated a five-times-higher number of H3K4me3-lost genes versus H3K4me3-gained ones. This epigenetic mark was significantly reduced near cadherin genes (from PCDHB8 to PCDHB15), which have an essential role in cell–cell adhesion by forming adherent junctions. Contrarily, H3K4me3 peaks were present at the locus of the homeobox genes (from HOXA2 to HOXA13) with a known role in regulating the transcriptional process. As H3K4me3 is an activating epigenetic mark, these findings could be further associated with invasive migration and dysregulation of Ras, PI3K-Akt, and MAPK pathways [58].

The equilibrium between histone H3 lysine 9 trimethylation (**H3K9me3**) and histone H3 lysine 9 acetylation (**H3K9ac**) plays a significant role in gene regulation and chromatin remodeling [59]. Several studies have investigated the role of H3K9me3 and H3K9ac in GBM. The specific equilibrium between these marks may vary in different contexts [60]. Some studies have reported a decrease in H3K9me3 levels in GBM cells compared to normal brain tissue, associated with transcriptional activation of oncogenes. Conversely, increased H3K9ac levels have been observed in GBM, suggesting a potential role in promoting tumor growth and survival [60,61].

Trimethylation of histone H3 lysine 27 (**H3K27me3**) in certain gene regions has been linked to poor prognosis in GBM, as increased levels of H3K27me3 have been observed [62]. This modification is associated with gene silencing and has been linked to suppressing tumor-suppressor genes, allowing for uncontrolled cell growth and proliferation [62].

H3K27me3 stain assessment was also proposed as a diagnostic procedure for more efficiently classifying diffuse glioma cases. The suggested diagnostic algorithm, based on combinatorial IDH1-, ATRX- and H3K27me3-positive immunostaining results, is described in detail in the article. Summarized data indicate that H3K27me3 loss and ATRX retention in IDH mutant cases strongly relate to oligodendrogliomas. In contrast, H3K27me3 retention with the same assayed parameters can be classified as oligodendrogliomas or astrocytoma, depending on positive or negative results of additional 1p/19q codeletion test, respectively [62]. Similar results regarding the same molecular hallmarks in oligodendroglioma were independently confirmed in the study conducted by Habiba et al. [63]. The study also evaluated cases of mutant IDH and 1p/19q codeleted oligodendrogliomas, mutant IDH astrocytoma, wild-type IDH astrocytoma, and wild-type IDH GBMs.

### 3.2. Histone Demethylases

The counterbalancing histone PTM is presented by histone demethylation, which removes methyl groups. Two families of histone demethylases have been distinguished: amino oxidase homolog lysine demethylases (KDMs) and JmjC domain-containing histone demethylases [64]. The role of these enzymes is also actively investigated in different tumor types, including GBM.

For example, an upregulated level of **JMJD2A** demethylase was attested in glioma tissues compared to normal brain tissues, associated with decreased levels of H3K9/H3K36 amino acids trimethylation. In vitro experiments demonstrated attenuated growth and colony formation in three lines of glioma cells (U251, T98G, and U87MG) after ectopic alleviation of JMJD2A expression, whereas the overexpression had contrary effects. The phosphoinositide-dependent kinase-1 (PDK1) expression regulation by the demethylase was presented as the underlying mechanism. The kinase, in turn, contributed to Akt-mTOR pathway activation and protein synthesis promotion, which directly influence cellular growth and proliferation. Additionally, performed rapamycin treatment presented a halted downstream activity of JMJD2A upon protein synthesis [65].

In another study, a histone H3K9 demethylase **KDM4C** was shown to contribute to both oncogene and tumor-suppressor gene regulation. Specifically, the analysis suggested that KDM4C downregulation attenuated cyclins’ (cyclin A1, D1, D2, and E2) and cyclin-dependent kinases’ (CDK2, CDK4, and CDK6) activities, events which could be related to evaluated inhibition of cell growth and viability. Meanwhile, the upregulation of pro-apoptotic (BAK, BAX, and PUMA) and downregulation of anti-apoptotic (BCL-XL) proteins suggested the implication of KDM4D in regulating apoptogenic effect. In addition to the overexpressed level of KDM4C, immunoblot analysis also revealed highly elevated levels of KDM4D and low or not-detected levels of KDM4A and KDM4B in GBM [66].

**KDM5A** and **UDX/KDM6B** lysine histone demethylases were presented as other pro-tumorigenic molecules with enhanced activity in TMZ-resistant GBM cells. In this regard, several synthetic inhibitors of KDM5A and KDM6B were investigated to determine their efficacy against developing malignant cells. The results indicated that GSK J4, an inhibitor of the KDM6 family, exhibited multiple anticancer effects, including inhibition of cell proliferation, blockage of the cell cycle at G1 and S phases, reduction of clonogenicity, and activation of apoptotic pathways. The authors also mentioned the lack of significant differences in the provided inhibitor’s effects between TMX-resistant and native cells. Finally, the study suggested the presence of synergic activity of GSK J4 and JIB 04 in cells that have acquired resistance to TMZ [67].

### 3.3. Regulation of Histone Acetyltransferases and Deacetylases by miRNAs

Another histone PTM includes histone acetylation, the process modulated by two types of enzymes: histone acetyltransferases (HATs) and histone deacetylases (HDACs), enzymes that participate in the addition and removal of acetyl groups, respectively. The altered expression of these enzymes is known to be a significant cause of deregulation in tumor-related pathways, including those from the nervous tissue [52]. Moreover, the link between methylation and acetylation of DNA and histones was noted in GBM and described in several studies, denoting the complex relationship of different epigenetic traits [45,60,68,69,70].

HATs regulate histone acetylation, a critical epigenetic modification that influences gene expression [70]. In the context of GBM, these enzymes have been studied for their role in tumor development and progression [70].

CREB-binding proteins (**CBP**) and **p300** are HATs that play essential roles in various cellular processes, including transcriptional regulation. They are frequently dysregulated in GBM and can contribute to tumor progression by promoting gene expression in cell growth and survival. RBBP4 interacts with p300 in GBM and modulates the expression of genes essential for cell survival [71].

**Tip60** regulates DNA repair and apoptosis. It was downregulated in GBM, and its loss may contribute to genomic instability and tumor development and progression [72].

On the other hand, histone deacetylases (HDACs) act by removing acetyl groups from histones, leading to gene transcription repression. Usually, HDACs inhibit oncogenes transcription and favor the overall healthy development of the cells. However, it is worth noting that in cancer, the intracellular landscape altered by dysregulated HDACs expression is very complex and connects a variety of cancer-associated proteins [73]. In this regard, in GBM, the terms **HDAC4**, **HDAC5**, **HDAC6**, and **HDAC11** were associated with anti-tumorigenic processes, whereas expression of **HDAC1** and HDAC3 had the opposite effect [74].

Despite this affirmation, **HDAC4** was attested overexpressed in glioma cell lines compared with adjacent non-tumor tissues and cell lines. It was positively associated with proliferative and invasive cell abilities. The results suggested that the enzyme was involved in downregulating p21 and p27 and upregulating CDK1 and CDK2 proteins, which are essential cell cycle regulators. Knockdown of HDAC4 led to cell cycle arrest at the G0/G1 phase and increased ROS levels. Furthermore, lower levels of E-cadherin and β-catenin and a higher level of vimentin were assayed in the HDAC4 overexpressing group, which concludes the importance of HDAC4 implication in cell invasiveness [75].

In the literature, HDAC4 was found to be targeted by miR-1 and miR-155 [76,77,78]. However, there are no studies regarding regulating HDAC4 by miRNAs in brain tumors. Apart from histone deacetylases, a connection was described between the upregulation of miR-155 and the downregulation of GABA-A receptor, which is targeted directly or indirectly by the miR-155 and was correlated with glioma grading [79].

In another example, the mRNA level of **HDAC1** was upregulated in glioma cell lines and glioma tissues compared to normal glial cell lines and non-neoplastic brain tissues. This event correlated with increased proliferation and invasion, as HDAC1 knockdown led to repression of these cell capabilities. Further analysis suggested that the deacetylase could positively regulate phosphorylated AKT and ERK and activate PI3K/AKT and MEK/ERK signaling pathways in vitro and in vivo [80]. Another study also supported the pro-tumorigenic activity of HDAC1 and demonstrated its implication in epithelial–mesenchymal transition and cell invasion. Functional enrichment analysis is also associated with HDAC1 overexpression and regulation of critical cellular pathways such as glycolysis, hypoxia, and inflammation [81].

Inhibition of **HDAC1/2** and activation of mitochondrial ClpP protease induced synergistic decrease of viability in GBM model systems. Alteration in tricarboxylic acid cycle activity and cell respiration is described as a possible mechanism alongside apoptosis activation [82]. Given this, mechanisms of HDAC1 inhibition became prominently sought. Although miR-449 and miR-874 have been shown to target HDAC1 in various cancer types, their implication in the enzyme’s regulation in GBM has not been widely explored yet [83,84,85,86,87].

A specific family of NAD^+^-dependent deacetylases is presented by sirtuins (SIRTs). They remove acetyl groups from histone proteins and other substrates, leading to transcriptional repression and chromatin compaction [70]. SIRTs have been implicated in various cellular processes, including ageing, metabolism, and cancer [70,88,89].

For example, SIRT1 is linked to multiple aspects of cancer biology. GBM was found to be overexpressed and associated with a poor prognosis due to tumor promotion, angiogenesis, and resistance to therapy [90]. In contrast, SIRT2 expression is frequently reduced, and its silencing may contribute to tumor progression via TP73 [91].

The study conducted by Chen and collaborators presented miR-22 as anti-oncogenic RNA found downregulated in GBM tissues and cells. It showed inhibitory activity toward SIRT1, EGFR, and metallopeptidase 9 (MMP9), proteins involved in cell survival, cell division, and basement membrane destruction. As a result, this activity dwindled cell proliferation, motility, and invasion of human GBM U87 and U251 cells [92].

An indirect inhibition of **SIRT1** was exhibited by miR-3908, which acted through AdipoR1 downregulation. The expression of AdipoR1 and its downstream AMPK/SIRT1 pathway proteins, or STAT2, suggests that miR-3908 contributed to regulating the AdipoR1/AMPK/SIRT1 signaling pathway. STAT2 was also found inhibited after miR-3908 overexpression. The results indicated that by inhibiting the paths above, miRNAs could induce suppression of cancer progression and GBM tumorigenicity [22].

A specific role in regulating SIRT1 during gliomagenesis and progression was also assigned to **miRNA-34a**, **miR-132,** and **miR-217** RNAs, which were shown to modulate the deacetylase [23].

Another study presented **miR-181a** as a potential anti-oncogene. This miR expression level was downregulated in glioma tissues and cell lines compared to normal brain tissues and gliocytes. Bioinformatics analysis revealed that miR-181a could target SIRT1, which was confirmed by Western blot analysis when overexpression of miR-181a and inhibition of SIRT1 were assayed. Moreover, **hsa_circ_0076248** was identified as sponging RNA for miR-181a, favoring SIRT1 overexpression, respectively. Downregulation of the hsa_circ_0076248 or upregulation of miR-181a presented anti-tumorigenic effects in glioma cells, specifically by inhibiting proliferation, invasion, and migration, as well as sensitizing the cells to TMZ treatment [93].

As NAD-dependent enzymes, dysregulated expression of SIRTs in GMB can be linked to the elevated level of nicotinamide phosphoribosyl transferase (NAMPT), an enzyme that catalyzes the first step in the biosynthesis of NAD [94]. NAMPT was found overexpressed in GBM, as well as in other human malignant tumors [95]. Moreover, macrovesicle-mediated transfer of the metabolic enzyme NAMPT was described as a novel mechanism for radioresistance provided by GSCs [96]. However, more investigations should be conducted to clarify the relationship between NAMPT’s and SIRT’s expression levels.

### 3.4. Other Histone PTMs (Phosphorylation, Ubiquitination)

Histone phosphorylation is a post-translational modification of histone proteins. While histone phosphorylation has been extensively studied in various biological processes, including gene regulation and DNA damage response, its specific role in GBM is still at the beginning of this field of research [97].

Increased levels of H3S10 phosphorylation (**H3S10ph**) have been observed, and this modification has been associated with tumor progression and poor prognosis [98]. H3S10ph is linked to chromatin remodeling and gene activation and has been implicated in promoting cell proliferation and invasiveness in GBM cells. H3T3 phosphorylation (**H3T3ph**) may regulate gene expression in cell cycle control and epithelial–mesenchymal transition (EMT), contributing to GBM progression [98].

Histone proteins can also be ubiquitinated and deubiquitinated by enzymatic machinery, which includes ubiquitin-activating enzyme (E1), ubiquitin-conjugating enzyme (E2), a ubiquitin ligase (E3), and a deubiquitinating enzyme (DUB). In the literature, dysregulation of histone ubiquitination was described in different cancer types. Genes encoding histone E3 ubiquitin ligases and DUBs are frequently shown to be altered [99].

The examples above regarding histone PTM regulation by miRNAs are summarized in Table 2 and schematically presented in Figure 1B.

## 4. Long Non-Coding RNAs (lncRNAs) in GBM

LncRNAs are RNA molecules longer than 200 nucleotides that do not code for proteins but play critical regulatory roles in biological processes. Many studies revealed lncRNAs to be implicated in GBM development and progression. There is an intricate interplay between them and epigenetic modifications, which plays a crucial role in the molecular landscape of GBM. LncRNAs can interact with histone-modifying enzymes, contributing to the regulation of gene expression.

For example, HOX transcript antisense RNA(HOTAIR) is upregulated in GBM and is associated with poor prognosis. HOTAIR acts as an epigenetic regulator by interacting with chromatin-modifying complexes, such as polycomb-repressive complex 2 (PRC2) and lysine-specific demethylase 1 (LSD1) [101]. HOTAIR can influence the chromatin state and gene expression patterns in GBM cells through these interactions. By recruiting LSD1, HOTAIR contributes to the repression of gene expression in GBM cells [102]. PTMs of histones also play a key role in regulating chromatin accessibility, influencing the expression of lncRNAs [103]. In glioblastoma, the HOTAIR gene locus can undergo histone modifications, such as increased levels of H3K27me3. The enrichment of H3K27me3 at the HOTAIR locus contributes to the repression of HOTAIR expression, influencing downstream gene expression patterns modifications [104].

Another representative lncRNA is MALAT1 that can physically interact with EZH2 [105]. This interaction may contribute to the recruitment of EZH2 to specific genomic loci, influencing the epigenetic landscape and gene expression in glioblastoma [106].

Several studies have demonstrated that **SNHG12** is upregulated in GBM cells resistant to TMZ. Increased expression of SNHG12 has been associated with decreased sensitivity to TMZ treatment [107]. Higher expression levels of SNHG12 have been associated with poor clinical outcomes and reduced overall survival in GBM patients receiving TMZ treatment. SNHG12 expression levels may be a potential biomarker for predicting TMZ resistance and patient prognosis [107].

In the study by Zheng, the expression level of radioresistance-associated long intergenic non-coding RNA 1 (**linc-RA1**) was increased in glioma cells and glioma tissue samples. Further analysis revealed that the RNA regulated the level of H2B K120 monoubiquitination by combining with the histone protein, thus inhibiting its interaction with ubiquitin-specific protease 44 (USP44). This event contributed to the inhibition of autophagy and favored cell radioresistance acquirement in vivo and in vitro [108].

In another example, the expression level of lncRNA **LINC00461** was significantly overexpressed in stem-like/treatment-resistant GBM cells. Analysis suggested that upstream molecular mechanisms could comprise upregulation of HDAC6 and its binding with an RNA-binding protein—carbon catabolite repression-negative on TATA-less (CCR4-NOT) core exoribonuclease subunit 6 (CNOT6), with a known role in RNA decay. Based on the prediction of the lncRNA–miRNA–mRNA networks, downstream mechanisms presented that the lncRNA could act as a sponge of **miR-485-3p**, which led to a lack of inhibition of maternal embryonic leucine zipper kinase (MELK) and minichromosomal maintenance protein 10 (MCM10). The results suggested that the LINC00461 increase could favor cell proliferation through cell cycle progression in GBM [109].

In contrast with the previous studies, **AC016405.3** is another lncRNA whose expression level was diminished in GBM. Acting as an anti-oncogene, its downregulation was associated with the advantage of proliferative and invasive cell abilities. The mechanism implied modulation of TET2 by lack of miRNA-19a-5p sponging [49].

Examples regarding epigenetic dysregulation caused by lncRNAs presented above are summarized in Table 3. Figure 1C depicts a representation of molecular mechanisms involved in lncRNA levels alteration.

Understanding how lncRNAs and epigenetic signatures interact provides insights into the complex regulatory networks governing gene expression. This knowledge is crucial for unraveling the mechanisms underlying diseases and developing targeted therapeutic interventions that modulate these regulatory pathways.

## 5. Exosomes in GBM

Exosomes are small membrane-bound vesicles secreted by cells into the extracellular environment, playing a crucial role in cell-to-cell communication. Exosomes can carry various types of cargo, including proteins, lipids, and nucleic acids, such as DNA, RNA, and microRNAs [110,111].

GBM-derived exosomes can influence the tumor microenvironment by modulating immune responses, angiogenesis, and stromal cell behavior [112]. Exosomes released by these cells contain specific molecular signatures or biomarkers that reflect the disease’s presence or status. Detecting and studying these exosomal signatures could have diagnostic, prognostic, or therapeutic implications in understanding and managing GBM [112].

Exosomal miRNAs from GBM can be transferred to recipient cells, affecting their gene expression and potentially contributing to tumorigenesis or altering the epigenetic landscape. In this regard, glioma cells can release exosomes containing specific microRNAs, including miR-1298-5p. The expression level of this transcript was downregulated in primary GMB cells, compared to normal human astrocytes in vitro, and it also was found to be released within cerebrospinal fluid exosomes in vivo. While experimentally overexpressed, miR-1298-5p could suppress glioma progression via SETD7 targeting and diminish tumor growth in vitro and in vivo. On the other hand, ectopically increased levels of the miRNA in myeloid-derived-suppressor cells (MDSCs) promoted the immunosuppressive effects via the NF-κB pathway. These findings suggested that glioma could achieve a double benefit from eliminating miR-1298-5p by lowering its intracellular content and targeting tumor-associated immune cells at the same time [113].

A recent study revealed that the presence of **miR-27b-3p** delivered by M2-like tumor-associated macrophages (TAMs) via exosomes contributes to the maintenance of this specific subset of cancer cells within the tumor. This implies that miR-27b-3p may support the survival, self-renewal, or other characteristics associated with GBM stem-like cells [114].

GBM exosomes may carry histone-modifying enzymes or histone marks that can impact the epigenetic state of target cells. For example, exosomal HDACs can influence the chromatin structure and gene expression in recipient cells [115,116]. Research in this area is ongoing, and understanding how epigenetic alterations within GBM-derived exosomes affect tumor progression, treatment resistance, and communication with distant organs is of great interest. It is worth noting that the field of exosome research has evolved rapidly, and new findings and insights may have emerged since my last update.

## 6. Conclusions

These epigenetic markers and modifications provide insights into the molecular mechanisms underlying GBM tumor development and progression and may have implications for diagnosis, prognosis, and treatment. These changes can be both causative and consequential to other inter- and intracellular processes, and the rationality of specific epigenetic modifications’ onset in GBM and their differential status in different cancer types is difficult to elucidate. Nevertheless, researchers continue to investigate the functional consequences of these epigenetic alterations and their therapeutic potential for improving outcomes in GBM patients.

The study of ncRNAs’ impact on regulating epigenetics machinery in GBM holds promise for developing new diagnostic tools and therapeutic strategies. By identifying specific miRNA expression profiles correlated with epigenetic alterations, researchers aim to understand glioblastoma’s underlying mechanisms better and potentially develop targeted therapies that can modulate these molecular changes to improve patient outcomes. Also, epigenetic changes can impact the regulation of GSCs, which are thought to play a significant role in the maintenance and recurrence of the disease.

In summary, studying epigenetic markers and modifications in GBM provides valuable insights into the molecular mechanisms driving the disease. Understanding these epigenetic alterations contributes to our knowledge of GBM pathogenesis, facilitates the development of biomarkers, and holds promise for developing targeted therapies to reverse aberrant epigenetic patterns.

## Figures and Tables

**Table 1:** miRNAs dysregulation and its effects on DNA epigenetic alteration machinery in GBM.

Brain Tumor Type	miRNA	Epigenetic Target	Observations	Ref.
Glioma	miR-185 ↑	DNMT-1	Affect global DNA methylation and induced the expression of the promoter-hypermethylated key genes (ANKDD1A/GAD1/HIST1H3E/ PCDHA8/CDHA13/PHOX2B/SIX3/SST)	[30]
GBM	miR-152-3p ↓	DNMT1 and methylation of NF2	Regulation of human glioma cell apoptosis and invasion via miR-152-3p	[37]
Glioma	miR-148a ↓	DNMT1	Tumor-suppressive miR-148a is silenced by CpG island hypermethylation in IDH1 mutant gliomas.	[40]
GBM	miR-29c ↓	DNMT3a/DNMT3b	Target MGMT, predict response to temozolomide	[39]
GBM	miR-129-5p ↓	DNMT3A	DNMT3A and miR-129-5p prognosis factors and therapeutic targets	[39]
GBM	miR-10b-5p ↑	TET2	SOX2/miR-10b-5p/TET2 axis that ↓ TET2 expression, ↓ 5hmC, ↑ 5mC levels, and induces stem cell features and tumor progression	[50]

↓—downregulation; ↑—upregulation.

**Table 2:** miRNAs dysregulation and its effect on histone PTMs in GBM.

Cancer Type	miRNA	Epigenetic Target	Observations	Ref.
GBM	miR-101-3p ↓	EZH2/ H3K27me3	Therapeutic strategy to target proliferation, migration, and angiogenesis	[54]
Glioma	miR-137 ↓	EZH2	Cell proliferation, invasion, and migration	[55]
Glioma	miR-524 ↓	EZH2	Therapeutic target, drug resistance	[100]
Glioma	miR-324-5p ↓	EZH2	Therapeutic target, drug resistance	[100]
GBM cells	miR-22 ↓	SIRT1	Cell proliferation, motility, and invasion	[92]
GBM	miR-3908 ↓	SIRT1	Regulation of AdipoR1/AMPK/SIRT1 signaling pathway; cancer progression and GBM tumorigenicity;	[22]
Glioma	miR-181a ↓	SIRT1	Inhibited by hsa_circ_0076248; cell proliferation, invasion, and migration, TMZ resistance	[93]

↓—downregulation.

**Table 3:** Examples of lncRNAs dysregulation in GBM.

Biological Material	lncRNA	Epigenetic Target	Observations	Ref.
GBM tissue specimens and cell lines	AC016405.3 ↓	DNA methylation and TET enzymes	Tumor suppressor role by regulation of TET2 via microRNA-19a-5p	[49]
GBM cells	HOTAIR ↑	Chromatin-modifying complexes, PRC2	Regulate cell cycle progression through EZH2	[101]
GBM cells	HOTAIR ↑	Histone demethylase enzyme, LSD1	Regulate cell cycle processes and induce apoptosis vis EZH2	[102]
GBM cells	SNHG12 ↑	DNA methylation MGMT	Temozolomide resistance; prognostic marker	[107]
Glioma cells	linc-RA1 ↑	Levels of H2BK120Ub1	Contribution to glioma radioresistance	[108]
Stem-like/treatment-resistant GBM cells	LINC00461 ↑	miR-485-3p	Upregulation of MELK and MCM10; cell cycle regulation.	[109]

↓—downregulation; ↑—upregulation.

## Data Availability

No new data were created or analyzed in this study. Data sharing is not applicable to this article.
